# Role of Private Enterprise in Cancer Control in Low to Middle Income Countries

**DOI:** 10.1155/2016/7121527

**Published:** 2016-12-13

**Authors:** Chukwumere E. Nwogu, Martin Mahoney, Ifeoma Okoye, Kenneth Ejiogu, Saby George, Grace Dy, Mutiu Jimoh, Omolola Salako, Oge Ilegbune, Bindiya Chugani, Emmanuel Ezeome, Abiodun O. Popoola, Arthur M. Michalek

**Affiliations:** ^1^Department of Thoracic Surgery, Roswell Park Cancer Institute, Buffalo, NY, USA; ^2^Department of Surgery, State University of New York, Buffalo, NY, USA; ^3^Lakeshore Cancer Center, Lagos, Nigeria; ^4^Department of Medicine, Roswell Park Cancer Institute, Buffalo, NY, USA; ^5^College of Medicine, University of Nigeria, Enugu, Nigeria; ^6^Lagos University Teaching Hospital, Lagos, Nigeria; ^7^Lagos State University Teaching Hospital, Lagos, Nigeria; ^8^Department of Public Health and Health Professions, State University of New York, Buffalo, NY, USA

## Abstract

*Background.* About 65% of cancer deaths globally occur in low to middle income countries (LMICs) where prioritization and allocation of resources to cancer care are often quite poor. In the absence of governmental focus on this problem, public-private partnerships may be an avenue to provide effective cancer control.* Methods.* This manuscript highlights the establishment of a nongovernmental organization (NGO) to stimulate the development of partnerships between oncology professionals, private enterprise, and academic institutions, both locally and internationally. Examples of capacity building, grant support, establishment of collaborative networks, and the development of a facility to provide clinical care are highlighted.* Results.* Collaborations were established between oncology professionals at academic institutions in the US and Nigeria. Cancer control workshops were conducted in Nigeria with grant support from the Union for International Cancer Control (UICC). A monthly tumor board conference was established at LASUTH in Lagos, and further capacity building is underway with grant support from the United States NCI. An outpatient, privately funded oncology clinic in Lagos has been launched.* Conclusion.* In LMICs, effective partnership between public and private institutions can lead to tangible strides in cancer control. The use of creative healthcare financing models can also support positive change.

## 1. Background 

The incidence of cancer is rising more rapidly in developing rather than industrialized countries. While 14.1 million new cancer cases and 8.2 million cancer deaths were reported worldwide in 2012, 57% (8 million) of new cancer cases and 65% (5.3 million) of the cancer deaths occurred in the less developed countries [1]. Low to middle income countries (LMICs) bear a disproportionate burden of cancer related deaths because they are less prepared to combat the disease [2]. Public academic institutions in such countries often have qualified individuals who are committed to improving these dismal statistics. However, the resources available for such efforts are markedly limited, centrally controlled, and given low priority. The solution to the challenge of providing affordable and effective care will not be simple nor will it be solved by a single initiative.

The key will lie in a multitude of efforts and initiatives. Rather than totally relying on the central government, public-private partnerships may contribute significantly to effective cancer control efforts. The aim of this descriptive report is to highlight the development of one such initiative in Nigeria, which is a lower middle income country [3] and lacks a comprehensive cancer center anywhere in the entire country. Such an institution would be able to serve as a focal point for the provision of clinical care, training of medical and ancillary personnel, and the coordination of research efforts. Clinical services offered at this center would include cancer screening, diagnostic imaging/interventions, surgical, medical, and radiation oncology treatment, and palliative care. Thus, the primary objective of this facility would be to translate academic cancer control knowledge to clinical application at the community level. Some aspects of the strategy employed in approaching the enormous cancer problem in Nigeria may be applicable to other countries.

## 2. Methods

A nongovernmental organization (NGO) was created in Lagos, Nigeria, by individuals interested in enhancing cancer care with the goal of stimulating the development of broad partnerships between oncology professionals, private enterprise, and academic institutions, locally and internationally. The founding objectives of this organization were to promote capacity building, establish collaborative networks, and develop a facility to provide clinical care. University teaching hospitals and nongovernmental organizations willing to participate as collaborative partners were identified.

Cancer control workshops were organized with support from the Union for International Cancer Control (UICC) [4, 5]. These workshops were seen as a cost-effective method to commence capacity building. Preliminary work for these workshops resulted in the development of memoranda of understanding signed between organizations in Nigeria and the United States to facilitate cooperative program development. Two workshops were provided, one in Enugu in 2009 [4] and the other in Lagos in 2013 [5], which consisted of didactic presentations, case studies, and break-out sessions which facilitated the exchange of ideas. Several needs were identified to be common across the two workshops and one in particular was the development of a center solely focused on cancer care.

Realizing the importance of the government in healthcare delivery, attempts were made to secure the commitment of the federal government and subsequently two state governments to fund the establishment of a cancer center. When these efforts failed to yield tangible results, a decision was made to seek private funding for the development of a facility focusing on early diagnosis of cancers and the provision of selected clinical services. Given the magnitude of the task, the project was broken up into several phases:

- 1st phase: outpatient cancer clinic,
- 2nd phase: clinical cancer center,
- 3rd phase: comprehensive cancer center,
- 4th phase: establishment of cancer centers in other cities.

## 3. Results

The initial nongovernmental organization that was established in response to the Nigerian cancer challenge was the Foundation for Cancer Care in West Africa (FCCWA). It is located in Lagos, Nigeria, which is the largest city in the subcontinent. It is led by a team of medical and nonmedical volunteers with broad academic and community ties. Thus, collaborative engagement was the central strategy for building alliances. Another NGO that was very important in the early activities of the Foundation was the “Breast without Spot” (BWS) Foundation, led by an accomplished academic physician and based in Enugu, Nigeria. Its activities focus on cancer control advocacy and quickly expanded from just one to 21 of the 36 states in Nigeria. FCCWA and BWS collaborated in facilitating the initial cancer control workshop in 2009 and the partnership has extended to cancer awareness events in other parts of the country and an effort to deploy community health educators to decrease late presentation of breast cancer patients.

Collaborations were established between oncology professionals at the Roswell Park Cancer Institute (RPCI) in Buffalo, NY, USA; the University of Nigeria Teaching Hospital (UNTH) in Enugu, Nigeria; and the Lagos State University Teaching Hospital (LASUTH) in Lagos, Nigeria. RPCI is the oldest comprehensive cancer center in the United States and is dedicated to cancer prevention, clinical care, and translational research. It receives support from the state of New York and from the United States National Cancer Institute. UNTH is a federal government tertiary medical center that is partnered with the University of Nigeria College of Medicine. Similarly, LASUTH is a state government tertiary medical center that is partnered with the Lagos State University College of Medicine. Cancer control workshops were conducted in Enugu in 2009 and in Lagos in 2013 with grant support from the Union for International Cancer Control (UICC) [4, 5] and involved teaching faculty from the US and from Nigeria with opportunities to learn from one another. One of the primary needs expressed at these workshops was the desire for continued communications and consultations with health professionals in the United States. Therefore, a monthly tumor board conference was established at LASUTH where both prospective and retrospective cases could be discussed amongst a vast spectrum of clinicians. For the initial several months, there was remote participation by one or more subspecialty oncologists from the Roswell Park Cancer Institute, depending on the types of cases that were being discussed. These oncologists were invited to give input and make recommendations. Several participants in the two workshops described above continued to collaborate extensively. An informal consortium was formed which resulted in interinstitutional academic faculty visits, joint manuscript publications, and grant applications. In partnership with other investigators at Roswell Park Cancer Institute and at the Noguchi Memorial Medical Research Institute, Accra, Ghana, funding has been obtained from the US National Cancer Institute to develop a well-trained cadre of oncology researchers in Nigeria and Ghana. The initial effort involved a focused breast, prostate, and cervical cancer workshop in Accra, Ghana, in May 2015 followed by the hosting of several participants at the Roswell Park Cancer Institute (RPCI) in Buffalo, NY, for further training.

**Figure 1:**
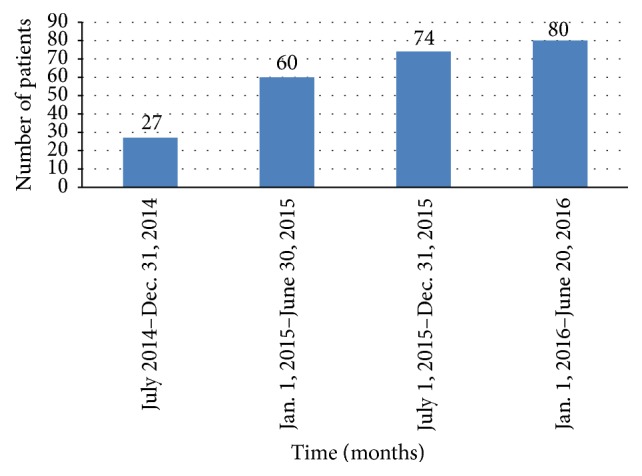
Number of new patients evaluated.

Another overlapping concern expressed by health professionals who attended the workshops was the need for a comprehensive clinical cancer center that could provide early diagnosis and effective, affordable cancer therapies. Given the identified need to establish a cancer center in Nigeria, FCCWA and RPCI signed a memorandum of understanding to do so. In the first phase of this effort, an outpatient oncology clinic—the Lakeshore Cancer Center—was set up in July 2014 and officially launched in January 2015. Services provided include cancer screening, diagnostic imaging and biopsies, outpatient surgery, chemotherapy administration, counseling, and palliative care. This clinic was begun in a rented 250-square meter facility, equipped with mammography, computed tomography (CT), a laboratory for relevant tests such as prostate specific antigen (PSA) and other assays and an ambulatory operating room for biopsies, lumpectomies, and basic gynecologic procedures. It initially was staffed by ten individuals including a medical officer, nurses, allied health professionals, and a small administrative team with reliance on telemedicine for all the oncology consultations. The staffing rapidly grew to twenty-five (25) individuals including domestically trained oncologists, a palliative care physician, a general medical practitioner, medical officers, nurses, a pharmacist, a radiology technician, a laboratory technologist, and administrative personnel. Radiologic image interpretation is provided remotely from India and additional oncology expertise has been provided from the United States by using telemedicine. This includes physician-physician video consultations, patient-initiated second-opinion requests, and imaging/pathology reviews by specialists in the United States. The increase in clinical activity is reflected in the rising number of new patients evaluated (Figure 1). The scope of surgical services provided was initially limited to outpatient tissue biopsies and lumpectomies. Inpatient mastectomies for breast cancer were added and more recently minimally invasive laparoscopic and thoracoscopic procedures as well as radical abdominal oncologic resections have been accomplished successfully. Thus, the 2nd phase of development (small cancer center) has been accomplished.

This has all been privately funded and patients pay “out-of-pocket” or with employer assistance. Efforts are underway to establish corporate cancer screening programs for employees of mid-sized to large commercial entities and partnerships with health maintenance organizations (HMOs) to extend their health insurance plans to cover cancer screening and treatment.

The need for clinical collaborations is well recognized. Thus, Lakeshore Cancer Center has expanded its clinical network primarily from one university teaching hospital in Nigeria to five such hospitals in Nigeria and one in Ghana, a private cancer center in Ghana, and several large private hospitals in Lagos, Nigeria. Negotiations to add radiotherapy capability as a public-private joint venture with a federal government teaching hospital are at an advanced stage.

## 4. Discussion

Cancer is a major global health challenge especially in low to middle income countries (LMICs). The increasing burden of cancer deaths in developing countries is largely due to the increased growth and aging of the populations, combined with lower mortality from infectious diseases and increased cancer associated risk factors, including changes in lifestyle choices, diets, and behaviors [6, 7]. Cancer mortality is also higher in resource limited countries due to lack of access to healthcare and adequacy of care [7, 8]. Beaulieu et al. (2009) estimated the case fatality from cancer to be 75% in low income countries and 46% in high income countries [9]. WHO has estimated that the global cancer burden will rise from 10 million new cases per year in 2000 to 16 million in 2020, with 70% of these cases coming from resource limited countries [8]. Thus, the rising incidence of cancer can no longer be overlooked, and urgent government funding and public-private partnerships are needed to help control the growing cancer burden in developing countries. These programs will benefit not only the population at risk but also the economy of these countries since a large proportion of cancer victims are in the prime of their lives.

Collaboration with the private sector is crucial because of the benefit of bringing additional funds and excellent management skills to tackle this enormous problem.

Academia has a prominent role to play also. Research into regional risk factors, etiologic agents, and disease characteristics can lead to more effective solutions.

Comparative effectiveness studies and implementation science can translate current cancer control knowledge into regionally specific and culturally sensitive interventions.

The current system of healthcare delivery in Nigeria includes both government and private sector involvement. Current health care system in Nigeria has been well described by Olakunde [10]. Public sector involvement includes the overall health system run by the Federal Ministry of Health (FMOH), state ministries of health, and local government health departments. Private sector involvement includes for-profit clinics/hospitals, nongovernmental organizations, and traditional care providers. The FMOH is the overall health policy formulating body responsible for coordination. It also provides tertiary care through university teaching hospitals and other federal specialized medical centers. The total Nigerian health expenditure as a percentage of the gross domestic product in 2012 was 3.4%, which is well behind other African countries such as Ghana (5.2%), Tanzania (7.1%), and South Africa (8.9%) [11]. As in most LMICs, individual health care is covered by a combination of governmental support, out-of-pocket payments, and health insurance. The exact levels and ratios of these payments vary tremendously by region and socioeconomic status of the individual. Individuals employed in certain governmental and private sectors may enjoy health insurance which provides access at an affordable cost while the majority of the population would rely on government provided health care which may not fully address patient needs and incur significant out-of-pocket costs. Out-of-pocket costs account for the highest proportion of healthcare expenditures. It is estimated that 64% of total health expenditures in Nigeria are covered by out-of-pocket payments [11]. Thus, the onus for healthcare is squarely placed upon the family. Such expenditures may be well out of reach of the average citizen and thus cause them to delay or neglect healthcare needs of their family. The solution to the provision of affordable and effective healthcare in Nigeria is a very complex issue. It cannot be solely solved by the government or by the private sector. The solution will involve a complex and collaborative interchange between governmental and nongovernmental organizations. The key foci of these initiatives will be on access to care and the ability to provide effective treatments.  Table 1 outlines some features of public versus private provision of cancer services. A hybrid public-private model may better capture the efficiency of private facilities while maintaining greater affordability and access that public facilities offer. This model has been successfully applied to advanced laboratory services at the University College Hospital (UCH), Ibadan, and the Lagos University Teaching Hospital, Nigeria.

The translation of cancer control knowledge from the academic realm to practical, community-level application requires immense collaborative efforts. In LMICs, government support of medical institutions is often limited by political will, bureaucracy, and competing demands for financial resources. There are many government officials that understand the benefits of cancer control. However, there is often inherent inertia in developing new programs. This can be frustrating or completely crippling. This was our experience in promoting a clinical cancer facility at the state government level. In such circumstances, it may become necessary for private enterprise to take the lead to break the impasse while establishing collaborations with academic institutions. As such privately funded, collaborative medical facilities become successful, it is probable that governmental agencies will be more motivated to participate. Given the novelty and magnitude of such a project in Nigeria, the execution was broken up into 3 phases—an outpatient clinic, a small inpatient facility, and then a comprehensive cancer center with widespread satellite clinics.

The most immediate future plans of this collaborative consortium in Nigeria include the training of a broad-spectrum of clinical and research personnel. This will involve academic exchanges between institutions in Nigeria and the United States.

Exchanges will first focus on transmission of best practices related to clinical care and then extend to areas of clinical research with an emphasis on implementation science.

The current clinical care facility has plans for expansion to a more multifaceted cancer center, including the development of the full spectrum of outpatient and inpatient services. This will require creative healthcare financing models, especially given the capital intensive nature of surgical and radiation facilities. The use of creative healthcare financing models can serve as a catalyst to effect positive change and lead to tangible strides in cancer control [12]. Academic knowledge can be harnessed from universities while effective management can be led by the private sector.

## Figures and Tables

**Table 1:** Public versus private cancer care.

	Traditional government cancer care	Private cancer center care
Location	Within large tertiary university teaching hospitals	In a facility dedicated solely to cancer care
Focus	Spread across multiple specialties including general medicine	Specifically on oncology
Scope of services	Very broad	Limited to oncology related services
Maintenance of facilities	Generally challenging	Easier to accomplish
Quality	Limited by local expertise	Enhanced by international telemedicine
Administrative model	Large, bureaucratic	Small, nimble
